# *Amblyomma sculptum* ticks on a giant otter from the Brazilian Pantanal

**DOI:** 10.1590/S1984-29612023053

**Published:** 2023-10-20

**Authors:** Grazielle Soresini, Nathalie Foerster, Fernando Paiva, Guilherme Mourão, Caroline Leuchtenberger

**Affiliations:** 1 Giant Otter Conservation Fund, Arroio do Meio, RS, Brasil; 2 Programa de Pós-Graduação em Ecologia e Conservação, Centro de Ciências Biológicas e da Saúde, Universidade Federal de Mato Grosso do Sul – UFMS, Campo Grande, MS, Brasil; 3 Instituto de Biociências, Universidade Federal de Mato Grosso do Sul – UFMS, Campo Grande, MS, Brasil; 4 Laboratório de Vida Selvagem, Embrapa Pantanal, Corumbá, MS, Brasil; 5 Instituto Federal Farroupilha – IFFAR, Santa Maria, RS, Brasil

**Keywords:** Ixodidae, Lutrinae, Mustelid, Pteronura brasiliensis, wild animals, Ixodidae, Lutrinae, Mustelídeo, Pteronura brasiliensis, animais selvagens

## Abstract

The giant otter (*Pteronura brasiliensis*) is a semiaquatic carnivore and a top predator in the trophic chain, considered a sentinel of freshwater ecosystems. Ticks are common ectoparasites of worldwide distribution and potential vectors of diseases. In this study, we report the ectoparasitism by ticks on a giant otter carcass found during monitoring activity at the Negro River, that holds a viable population of this endangered species in the Brazilian Pantanal. A total of three tick specimens were collected: two adults were identified as *Amblyomma sculptum* and a nymph as *Amblyomma* spp. There is a lack of information about the health of free-ranging giant otters and this report contributes to elucidate some of the host-parasite relationships, although much more research is needed to expand the knowledge about which kinds of pathogens are circulating in the species, especially among those transmitted by ticks.

## Resumo

A ariranha (*Pteronura brasiliensis*) é um carnívoro semiaquático e um predador de topo na cadeia trófica, considerada sentinela dos ecossistemas de água doce. Os carrapatos são ectoparasitos comuns com distribuição mundial e potenciais vetores de doenças. Neste estudo, foi relatado o ectoparasitismo por carrapatos em uma carcaça de ariranha encontrada durante atividade de monitoramento no Rio Negro, no Pantanal brasileiro, que abriga uma população viável desta espécie ameaçada de extinção. Um total de três espécimes de carrapatos foram coletados: dois adultos foram identificados como *Amblyomma sculptum* e uma ninfa como *Amblyomma* spp. Há uma lacuna de informações sobre a saúde das ariranhas de vida livre e este relato contribui para elucidar algumas das relações parasita-hospedeiro, embora ainda seja necessária mais pesquisa para ampliar o conhecimento sobre quais tipos de patógenos estão circulando na espécie, especialmente entre os transmitidos por carrapatos.

## Introduction

The giant otter (*Pteronura brasiliensis*) is considered to be the largest river otter in the world. Giant otters live in highly cohesive groups and are globally endangered (Groenendijk et al., 2022). The species is endemic to South America and, despite its wide distribution across Bolivia, Brazil, Colombia, Ecuador, French Guiana, Guyana, Peru, Suriname and Venezuela, the species has lost almost 40% of its historical range. The populations of Argentina and Uruguay are practically extinct. In Paraguay, it has been reduced to a small subpopulation (Groenendijk et al., 2022). In Brazil, it currently occurs in the Amazon, in the Pantanal, and in a restricted portion of the Cerrado, corresponding to the Araguaia River Basin (Leuchtenberger et al., 2018).

Extensive research on the ecology and biology of this species has been carried out over recent decades, but the health condition of these animals in the wild is still poorly known. Additionally, their parasitism by ticks and the diseases caused by agents that ticks transmit among hosts are largely unknown. There are a few records in Brazil of ticks parasitizing Neotropical otters (*Lontra longicaudis*) in the states of Paraná, São Paulo, Minas Gerais and Santa Catarina (Arzua et al., 2005; Labruna et al., 2005; Martins et al., 2015; Verdin et al., 2015), in the Atlantic Rainforest and Cerrado ecoregions. However, until we know, there is just one study reporting the presence of a single tick on a giant otter caught in the Balbina hydroelectric reservoir, in the Brazilian Amazon region (Rosas et al., 2015). So far, there are no records for the Pantanal.

Ticks are among the most important vectors of pathogens that cause diseases in animals and humans. Cohabitation of domestic and wild animals in the same environment favors the spread of etiological agents to new environments and species. The Pantanal is a large Neotropical floodplain, and one of the most protected ecoregions in Brazil, which still has 80% of its native vegetation and harbors viable populations of several endangered vertebrates, such as the giant armadillo (*Priodontes maximus*) and the giant anteater (*Myrmecophaga tridactyla*). During the last century, high numbers of zebu cattle, horses and dogs were introduced in the Brazilian Pantanal (Pereira et al., 2000), which may have directly impacted the ticks associated with wildlife (Pereira et al., 2000; Cançado et al., 2008).

The Ixodidae family comprises the “hard ticks”, due to a large anterodorsal sclerite, a dorsal plate or scutum in all stages of biological development. There is a remarkable sexual dimorphism. In males, the posterior border of opisthosoma can present sclerotized dorsally structures called festoons (e.g. species of the genus *Amblyomma*) or ventral plates (e.g. species of the genus *Rhipicephalus*), also present in the Prostriata group (Barros-Battesti et al., 2006). The Ixodidae includes the Metastriata group with the subfamily Amblyomminae, with two genera: *Amblyomma* and *Archaeocroton* (sin. *Aponomma*). This last genus occurs in Australia while *Amblyomma* genus appears to have originated in Antarctica and subsequently dispersed to Australia (Beati & Klompen, 2019). The purpose of this study was to report on ectoparasitism by ticks collected from the carcass of a wild giant otter in the Brazilian Pantanal, the first record of ticks on a semiaquatic mustelid species in this seasonally flooding ecoregion.

## Methods

A giant otter individual was found dead at the Negro River (19°34’42” S, 56°09’11” W), in the southern Brazilian Pantanal region. The carcass, which showed no clear external signs of the cause of death, was collected in a fresh condition, transported to the city of Campo Grande and was frozen in the zoology laboratory of the Federal University of Mato Grosso do Sul for subsequent necropsy, which was performed in July 2021. This otter was an adult of length 170 cm and weight 20.65 kg, and it was identified as the dominant male of one of the groups monitored by our team in this river since 2019.

**Figure 1:**
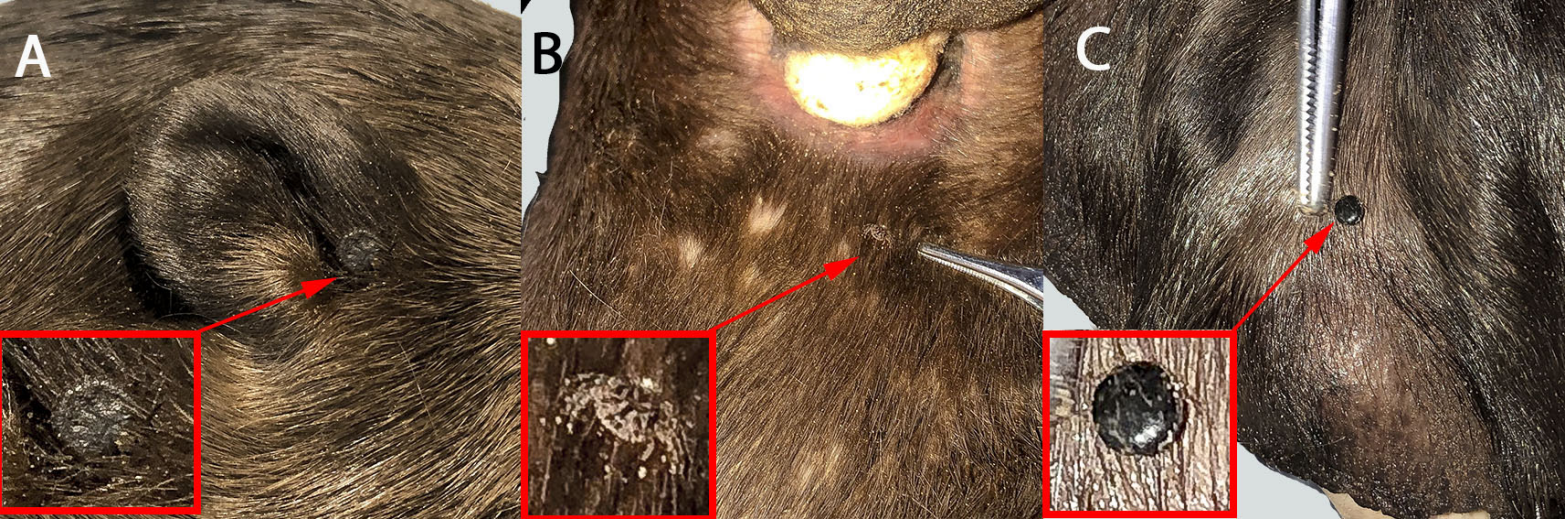
Ticks found attached to the carcass of a giant otter (*Pteronura brasiliensis*) that was recovered at the Negro River, in the southern part of the Brazilian Pantanal region: one female of *Amblyomma sculptum* on the ear (A) and one male on the tail (B). *Amblyomma* spp. nymph on the interdigital membrane of a thoracic limb (C).

During the external examination, after slowly unfreezing the carcass over a 12-hour period, the ticks found were manually removed and stored in plastic vials, containing 70 GL ethanol, for subsequent taxonomic identification. The site of tick attachment was recorded (Figure 1). The specimens were deposited in the Zoological Reference Collection of the Federal University of Mato Grosso do Sul, under the numbers ZUFMS-CHE00552 to CHE00554.

The ticks were identified using published morphological keys for ticks and corresponding literature (Guimarães et al., 2001; Barros-Battesti et al., 2006; Martins et al., 2010; 2016). The specimens were examined for their taxonomic characteristics by means of light microscopy, using a Leica M205 C™ stereomicroscope, equipped with a Leica camera, model DFC 420™ (Leica Microsystems™, Wetzlar and Mannheim, Germany), and images were registered using the Leica Application Suite image analysis software (LAS™ 3.8; Leica Microsystems™, Wetzlar and Mannheim, Germany).

**Figure 2:**
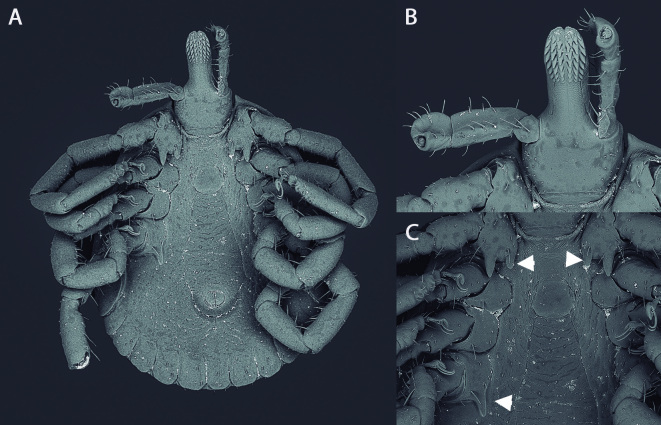
Scanning electron microscopy images of a male specimen of *Amblyomma sculptum* that was found attached to the carcass of a giant otter (*Pteronura brasiliensis*) that was recovered at the Negro River, in the southern part of the Brazilian Pantanal region: A- ventral view, B- detail of capitula and hypostome, and C- detail of coxae I-IV.

The specimen selected for scanning electron microscopy (SEM) (Figure 2) was dehydrated in a progressive ethyl alcohol series from 70 to 99 GL, with one-hour intervals between the dilutions (70, 80, 90 and 99 GL). Then, the specimen was immersed in hexamethyldisilazane (Cat. Number 440191; Sigma-Aldrich™) for 10 minutes and followed by deposition onto carbon conductive tabs, of 12 mm OD (PELCO Tabs™; Ted Pella®, Inc., USA). The tabs were glued to PELCO® Q pin stubs, of dimensions 12.7 x 12.7 mm (Ted Pella®, Inc., USA). The microscopy images were documented using a Hitachi® model TM3000™ scanning electron microscope (Hitachi, Tokyo, Japan), which was operated in the analysis mode.

## Results

A total of three ticks were obtained: one female located on the ear (Figure 1A; ZUFMS-CHE00552), one male on the tail (Figure 1B; ZUFMS-CHE00554) and one nymph on the interdigital membrane of a thoracic limb (Figure 1C; ZUFMS-CHE00553). The adult specimens were identified as *Amblyomma sculptum* Berlese, 1888 (formerly *Amblyomma cajennense*). We were not able to confirm the species of the nymph (*Amblyomma* spp.). Their taxonomic characteristics were:

Female: oval body, length from scapulae to posterior margin 4.55 x 4.06 mm; specimen without capitulum, dorsal shield subtriangular, brown, with whitish spots accentuating the central part, anal sulcus posterior to anus, rostrum long, with eyes present, coxa I with two unequal spurs, external longer than the internal one, which was blunt, coxa II-IV with a single triangular spur, spiracular plate in the shape of a comma.

Male: oval body, total body size 3.10 x 2.15 mm (Figure 2A); basis capituli subrectangular dorsally, scutum ornate, spatulate hypostome (Figure 2B), with formula 3/3, marginal groove complete and limiting the festoons, cervical spots elongated posteriorly with numerous punctuations, coxa I with two unequal spurs, the external covering the next article and the internal one shorter, coxa IV with a single spur longer than of the article, spiracular plate in the form of a comma (Figure 2C).

Nymph: oval body, total body size 3.95 x 2.76 mm; basis capituli rectangular dorsally, eyes in the medial-lateral portion of the scutum, hypostome with dental formula 2/2, coxa I with two pointed spurs, the external about twice as long as the internal; coxae II-IV with a small triangular spur. The specimen was quite engorged, and the final identification was not possible.

## Discussion

The taxonomic status of *A. cajennense* was reassessed and, according to the new classification, the *A. cajennense* species complex is currently represented by two species in Brazil: *A. cajennense sensu stricto* (*s.s*.) and *A. sculptum* (Estrada-Peña et al., 2014; Nava et al., 2014; Martins et al., 2016). The wide geographic distribution of *A. sculptum* in Brazil includes most of the Cerrado, the Pantanal, and Atlantic Forest ecoregions, while *A. cajennense* (*s*. *s*.) is restricted to the Amazonian region and transition areas with the Cerrado (Martins et al., 2016). Consequently, all records from the Brazilian Pantanal are considered to be *A. sculptum* (Martins et al., 2016; Cançado et al., 2017), the most prevalent tick species in the Pantanal (Pereira et al., 2000; Ramos et al., 2014; Kluyber et al., 2016; Cançado et al., 2017). Instead, the tick species previously reported on the giant otter by Rosas et al. (2015) in the Central Amazon is *A. cajennense s.s*.

Ticks are obligate hematophagous arthropods of significant importance to human and veterinary medicine, since they are vectors or reservoirs for transmission of pathogenic agents including bacteria, viruses, fungi and protozoa, during their feeding process on their hosts. Hard ticks parasitize several hosts (life cycles involving one to three hosts) and present some stages in the environment. They can transmit a broad range of pathogens worldwide. *Amblyomma sculptum* is considered epidemiologically important, since it is the main vector in Latin America of the bacterium *Rickettsia rickettsii*, that is the etiological agent of Brazilian spotted fever. This is a potentially fatal tick-borne disease of the New World (Martins et al., 2016; Dias et al., 2020) which was often associated with the proximity of horses or capybaras with humans (Dias et al., 2020). A number of other pathogens transmitted by ticks were also reported in various wild and domestic species in the Pantanal (e.g. Barros et al., 2015; Wolf et al., 2016; Dias et al., 2020).

Feral pigs, an alien introduced species, are considered to be very suitable hosts for this tick in that region (Ramos et al., 2014), with 100% prevalence and high infestation intensity (Cançado et al., 2017). Besides, many terrestrial mammals in the southern Pantanal are often infested by *A. sculptum* (e.g. Pereira et al., 2000; Kluyber et al., 2016; Cançado et al., 2017). However, the occurrence of *A. sculptum* parasitizing semiaquatic mustelids remains unknown in the Pantanal. To the date, the only other report of ectoparasitism in giant otters in the Pantanal was a myiasis caused by *Cochliomyia hominivorax* (Foerster et al., 2022). Findings of ectoparasites on giant otters are rare given this animal’s extensive grooming and allogrooming behavior, along with the difficulty of capturing this species or recovering carcasses in the wild.

Giant otters are semiaquatic, associated with freshwater habitats. They build dens along riverbanks and lakes and use communal latrines and campsites, where ticks can find good conditions for survival. On the other hand, this species’ usual pattern of frequent change of dens may prevent high infestations of ectoparasites (Schweizer, 1992).

Although tick infestation is rare in otters, the prevalence might be an important health issue. Studies on larger series of tick parasitism on otters are scarce (e.g. Rohner et al., 2022) and seem to be nonexistent with Brazilian species. To rely on carcasses to study ticks in otters from South America is not an exclusivity of the present study. For example, Verdin et al. (2015) also collected ticks (*Amblyomma ovale* Koch, 1844) from a carcass. Guimarães et al. (2001) cited *A. ovale* in Neotropical otter for several Brazilian states and Arzua et al. (2005) reported the presence of *A. ovale* in this species in Paraná state, while Verdin et al. (2015) collected *A. ovale* from the carcass of a Neotropical otter in the Atlantic Rainforest in the state of Santa Catarina, southern Brazil.

The variety of free-ranging species that are parasitized by *A. sculptum* makes its control virtually impracticable. Environmental control using acaricides would be inadequate because of the impact on regional biodiversity and contamination of water bodies (Cançado et al., 2017). In fact, water pollution is currently considered to be one of the main threats to giant otters in Brazil and throughout most of their range (Leuchtenberger et al., 2018).

Epidemiological studies on giant otters are still scarce in Brazil and the impact of pathogen circulation on the species is unknown. This report contributes to elucidating the host-parasite relationships, although much information is still needed.

Anthropogenic activities and invasion of the natural habitats of wild species such as the giant otter can have negative consequences not only for the wildlife, but also for the health of domestic animals and humans. This situation gives rise to important risks from a “one health” point of view, considering that parasites and infectious agents may cause disease in some species, using other hosts as reservoirs. The co-occurrence between domestic and wildlife are a matter of concern, once it can give rise to spillover of parasites/pathogens that can represent an important threat to endangered wildlife as well as to humans.
